# gga-miR-1603 and gga-miR-1794 directly target viral L gene and function as a broad-spectrum antiviral factor against NDV replication

**DOI:** 10.1080/21505594.2020.1864136

**Published:** 2020-12-29

**Authors:** Yu Chen, Shanshan Zhu, Jiao Hu, Zenglei Hu, Xiaowen Liu, Xiaoquan Wang, Min Gu, Shunlin Hu, Xiufan Liu

**Affiliations:** a Animal Infectious Disease Laboratory, College of Veterinary Medicine, Yangzhou University, Yangzhou, China; b Jiangsu Co-innovation Center for Prevention and Control of Important Animal Infectious Diseases and Zoonosis, Yangzhou University, Yangzhou, China; c Jiangsu Key Laboratory of Zoonosis, Yangzhou University, Yangzhou, China

**Keywords:** Newcastle disease virus, miRNAs, gga-miR-1603, gga-miR-1794, l gene

## Abstract

As the causative agent of Newcastle disease (ND), Newcastle disease virus (NDV) has seriously restricted the development of the poultry industry. Previous research has shown that miRNAs, members of the small noncoding RNA family, are implicated in the regulation NDV replication through extensive interactions with host mRNAs, but whether miRNAs affect NDV replication by directly binding to the NDV antigenome remains unclear. In this study, potential *Gallus gallus* miRNAs targeting the antigenome of NDV were bioinformatically predicted using the online software RegRNA 2.0, and gga-miR-1603 and gga-miR-1794 were identified as targeting the viral L gene directly through dual-luciferase reporter assays. Sequence alignment analysis demonstrated that multiple genotypes of NDVs harbored highly conserved binding sites for gga-miR-1603 and gga-miR-1794 in the viral antigenome located at 8611–8634 nt and 14,490–14,514 nt, respectively. Meanwhile, we found that gga-miR-1603 and gga-miR-1794 negatively regulated the expression of viral L gene at both the RNA and protein levels, as well as viral replication *in vitro*. Furthermore, NDV infection had no effect on endogenous gga-miR-1603 and gga-miR-1794 expression in various avian cell lines. Overall, our present study demonstrated that gga-miR-1603 and gga-miR-1794 directly bind to the viral L gene to facilitate ts degradation and inhibit the replication of multiple genotypes of NDVs *in vitro*. These findings will provide us with important clues for antiviral therapy against NDV infection.

## Introduction

Virulent Newcastle disease virus (NDV) can naturally infect almost all species of birds and cause Newcastle disease (ND), which seriously restricts the development of the poultry industry [1,2]. The genome of NDV is composed of a negative sense RNA, which is transcribed into a positive sense antigeome upon entry into host cells and is then translated into viral proteins [2]. Although NDV has only one serotype, they are nevertheless highly diverse genetically [3]. In line with an updated unified phylogenetic classification system [4], NDV isolates belong to two distinct categories, of which class I NDV strains are almost all avirulent within only one genotype, whereas class Ⅱ strains are diverse in virulence and can be separated into more than 20 genotypes.

As a member of the small non-coding RNAs family, numerous studies have shown that microRNAs (miRNAs) play an extensive regulatory role within a cell [5]. Since they were first discovered in the 1990s, more than 30,000 miRNAs have been identified in hundreds of species, among which, more than 1,200 miRNAs have been found in *Gallus gallus*. It was recognized that the miRNA “seed sequence” composed of the first 7–8 nt in the 5′ end is crucial for the interaction with mRNA [6]. Through base-pairing interactions with their mRNA targets, miRNAs function as important endogenous regulators of numerous processes within a cell through post-transcriptional translation inhibition or gene silencing [7]. Viral replication is completely dependent on the cells, and thus, the extensive regulatory effect of miRNA on host mRNAs is bound to influence viral replication in host cells. Our previous studies have revealed that NDV infection of DF-1 cells leads to expressional changes of dozens of miRNAs, among which two up-regulated miRNAs, gga-miR-451 and gga-miR-19b-3p, modulate NDV replication via interactions with different host mRNAs [8,9]. Besides NDV, the miRNA-mediated regulation of viral replication through interactions with host mRNAs is also found in many other viruses, such as influenza virus [10–12], avian leucosis virus J [13,14] and infectious bursal disease virus (IBDV) [15–17].

In addition to interactions with host mRNAs, evidence is accumulating that miRNAs can perform their functions of regulating the life cycles of certain viruses by directly binding their genomes [18–21]. On the one hand, the genome of a positive-strand RNA virus can be directly bound by miRNA due to its structural similarity to the host mRNA. For example, miR-181 suppresses porcine reproductive and respiratory syndrome virus (PRRSV) replication by specifically binding ORF4 [22]. Likewise, miR-378, miR-23, and miR-505 were proven to protect against PRRSV infection due to their ability to directly bind ORF7 of PRRSV genomic RNA [23]. On the other hand, miRNAs can also bind the positive-sense antigenome produced by the transcription of negative-strand RNA viruses. For example, miR-485, miR-323, miR-3145, miR-654 and miR-491 were identified as directly targeting the influenza virus PB1 gene, leading to the inhibition of viral replication *in vitro* [24–26]. Similar degradation of viral RNA is found when let-7 c binds the 3′ untranslated region (UTR) of the influenza virus M gene in A549 cells [27]. Although much attention has been paid to studying the interplay between miRNAs and viral infection, whether miRNAs can regulate NDV replication by directly targeting the NDV antigenome remains unknown.

In this study, an online software RegRNA 2.0 and dual-luciferase reporter assay were employed to screen and verify potential cellular miRNAs that might directly bind the antigenome of NDV. Gga-miR-1603 and gga-miR-1794 were found to directly interact with the L gene of NDV. Functional analysis indicated that both inhibited expression of the viral L gene at both the RNA and protein levels, as well as viral replication in DF-1 cells. To our knowledge, this study is the first to demonstrate that cellular miRNAs regulate NDV replication by targeting the viral L gene. The findings of this study suggest that gga-miR-1603 and gga-miR-1794 could serve as potential therapeutic targets to inhibit NDV infection.

## Materials and methods

### Cell lines and virus propagation

The immortalized chicken embryonic fibroblast cell line DF-1, chicken hepatocellular carcinoma cell line LMH, chicken macrophage-like cell line HD11, and chicken embryonic fibroblast (CEF) primary cells were maintained in Dulbecco’s modified Eagle’s medium (Invitrogen, USA) supplemented with 10% fetal bovine serum (FBS; Invitrogen, USA), at 37°C, in a 5% CO_2_ incubator. NDV strains LX, La Sota, Mukterswar, Herts/33, NDV-P05, Kuwait, JS 5/05, and ZJ1 were isolated and identified by our laboratory. The biological characteristics of these NDV isolates were determined in previous studies [28–32] and are shown in Table 1.

**Table 1.** Details of NDV strains used in this study

NDV strains	Genotype ^a^	Pathotype	Accession number
LX	I	Lentogenic	KF494201
La Sota	II	Lentogenic	AF077761
Mukterswar	III	Mesogenic	EF201805
Herts/33	IV	Velogenic	AY741404
NDV-P05	V	Velogenic	HM117720
Kuwait	ⅩⅩ (formerly Ⅵ ^b^)	Velogenic	MK978147
JS 5/05	Ⅶ	Velogenic	JN631747
ZJ1	Ⅶ	Velogenic	AF431744

^a^Genotyping was performed using an updated classification system as described previously [4].

^b^according to a previous genotyping method as described by Diel et al [47].

### Potential miRNAs prediction and conservation analysis

To investigate whether cellular miRNA can directly target NDV antigenomic RNA, potential *Gallus gallus* miRNA binding sites in the antigenomic RNAs of different NDV strains were predicted using RegRNA 2.0 software [33] (http://regrna2.mbc.nctu.edu.tw/) with filter criteria as follows: score ≥ 150.00 and minimum free energy (MFE) ≤ −25.00 Kcal/mol. The prediction results of different strains were intersected using Venny 2.1 (https://bioinfogp.cnb.csic.es/tools/venny/) and the overlaps were selected for conservation analysis. We aligned predicted miRNA binding sites in 192 NDV strains collected from GenBank using MEGA 5 software (DNAStar Inc. USA) and genotyping of these strains was performed using an updated classification system as described in our previous study [29].

### Plasmids constructions

The dual-luciferase vector pmiR-RB-reporter^TM^ (Ribobio, China), expressing *Renilla* and firefly luciferase simultaneously, was used as the parent vector for 3′ UTR reporter analysis experiments. The viral gene segments, including NP, P, F and HN, were PCR-amplified using the cDNA of the classical vaccine strain La Sota as a template and were then cloned into the pmiR-RB-reporter^TM^ vector. To ensure sufficient transfection efficiency, the L gene was divided into three segments and each was cloned into the pmiR-RB-reporter^TM^ vector. These constructed vectors were named pmiR-La Sota-NP, -P, -F, -HN, -L1, -L2, and -L3. To generate dual-luciferase plasmids containing L1 and L3 regions of different genotypes of NDV strains, the corresponding regions were amplified using the cDNA extracted from indicated strains as templates and cloned into the pmiR-RB-reporter^TM^ vector. These vectors were pmiR-LX-L1/L3, pmiR-Mukteswar-L1/L3, pmiR-Herts/33-L1/L3, pmiR-P05-L1/L3, pmiR-Kuwait-L1/L3, and pmiR-ZJ1-L1/L3. To generate mutant miRNA binding site reporter constructs, mutations were introduced into the viral L gene at the binding sites for gga-miR-1603 and gga-miR-1794 seed regions using a Fast Mutagenesis System Kit (Transgen Biotech, China) following the manufacturer’s protocol. The mutated vectors were named pmiR-La Sota-Mut-L1/L3, pmiR-LX-Mut-L1/L3, pmiR-Mukteswar-Mut-L1/L3, pmiR-Herts/33-Mut-L1/L3, pmiR-P05-Mut-L1/L3, pmiR-Kuwait-Mut-L1/L3, and pmiR-ZJ1-Mut-L1/L3. To generate pCMV-Flag-La L and pCMV-Flag-ZJ1 L, the L gene of La Sota and ZJ1 were cloned into the fusional expression vector pCMV-Flag (Beyotime, China). All plasmids constructed in this study were confirmed by sequencing, and the primers used are shown in Table S1.

### Transfection and quantitative real-time PCR (qRT-PCR) detection of miRNA oligonucleotides

The sequence of miRNA oligonucleotides (Genepharma Company, China), including miRNA mimics or inhibitors and corresponding negative control, are shown in Table S2. For miRNA transfection, miRNA oligonucleotides (100 nM) were transfected into DF-1 cells using EL Transfection Reagent (Transgen Biotech, China) following the protocol supplied. miRNA expression levels were determined using qRT-PCR as previously described [8]. Briefly, cellular miRNAs were extracted at indicated time points post-transfection or post-infection using the miRNA Extraction Kit (HaiGene, China). Then, cDNA was synthesized with the one Step miRNA cDNA Synthesis Kit (HaiGene, China). qRT-PCR was operated with the HG miRNA SYBR Green PCR Kit (HaiGene, China) on a determined 480 machine (Roche, Switzerland) with specific primers listed in Table 2. 5S rRNA was used as a reference gene for normalization of miRNAs using the 2^−ΔΔCt^ method.

**Table 2.** Primers used for miRNAs detection by qRT-PCR

Primers	Primer sequences (5ʹ-3ʹ)
gga-miR-551 F	GGCGACCCATACTTGGT’ GGTCCAGTTTTTTTTTTTTTTTCTGA
gga-miR-551 R	
gga-miR-1671 F	CAGGTGAGGACTGTTGAGT AGTTTTTTTTTTTTTTTGGCCACT
gga-miR-1671 R	
gga-miR-1574-5p F	AGCTGTGACTTCTCCTTGT TCCAGTTTTTTTTTTTTTTTCTGACA
gga-miR-1574-5p R	
gga-miR-1658-3p F	GCTGTGGGTTGGTGTTGA TCCAGTTTTTTTTTTTTTTTCCATCA
gga-miR-1658-3p R	
gga-miR-1597 F	GTGAGGAGCTCTGCAAG CAGTTTTTTTTTTTTTTTGCATGCT
gga-miR-1597 R	
gga-miR-1610 F	GTGGCTTGTGGTGGAAC GTTTTTTTTTTTTTTTCGCCCGTT
gga-miR-1610 R	
gga-miR-1735 F	GGCTTTGGGCAGCA GTCCAGTTTTTTTTTTTTTTTCAGATG
gga-miR-1735 R	
gga-miR-1695 F	AGCACAGTTTGGTCATGG GTCCAGTTTTTTTTTTTTTTTGCTC
gga-miR-1695 R	
gga-miR-460b-5p F	GTCCTCATTGTACATGCTGT TCCAGTTTTTTTTTTTTTTTCACACA
gga-miR-460b-5p R	
gga-miR-1603 F	GTGGTTGGTTTGGTGCT GTCCAGTTTTTTTTTTTTTTTGACAG
gga-miR-1603 R	
gga-miR-1794 F	GGCCAGAATGGACATGG GGTCCAGTTTTTTTTTTTTTTTGCT
gga-miR-1794 R	
5S rRNA F	AACGCCCGATCTCGTCTGAT AGTCTCCCATCCAAGTACTAACCG
5S rRNA R	

### Dual-luciferase reporter assay

DF-1 cells cultured on 12-well plate were co-transfected with indicated plasmids (250 ng) and miRNA oligonucleotides (100 nM) using EL Transfection Reagent (Transgen Biotech, China). After 24 h, luciferase activities were determined using the Dual-GLO® Luciferase Assay System Kit (Promega, USA) following the protocol supplied. The relative luciferase activities were calculated by dividing *Renilla* luciferase values by internal control firefly luciferase values.

### Western blotting

To determine whether gga-miR-1603 and gga-miR-1794 could affect viral L protein expression, DF-1 cells co-transfected with indicated RNA oligonucleotides and pCMV-Flag-La L or pCMV-Flag-ZJ1 L were lysed in RIPA lysis buffer (Beyotime, China) at 24 hours post-transfection. The protein samples with equal amounts were resolved by SDS-PAGE and then transferred onto polyvinylidene difluoride membranes. After blocking in 3% skim milk at 37°C for 1 h, the membranes were incubated with appropriate primary antibodies at 4°C overnight followed by incubation with HRP-conjugated secondary antibodies at 37°C for 1 h. The secondary antibodies, mouse monoclonal anti-Flag and anti-β-actin were all purchased from Transgene, China.

### Virus infection and determination of virus titers

To detect whether NDV infection regulates gga-miR-1604 and gga-miR-1794 expression, DF-1 cells at 80–90% conﬂuence were inoculated with indicated NDV isolates at a multiplicity of infection (MOI) of 0.1. The expression levels of gga-miR-1604 and gga-miR-1794 were detected at indicated time points post-infection by miRNA qRT-PCR as described previously herein. To explore the effect of gga-miR-1604 and gga-miR-1794 on NDV replication, DF-1 cells were first transfected with indicated miRNA oligonucleotides for 24 h and then the cells were inoculated with indicated NDV isolates (0.1 MOI). The culture supernatants were collected at indicated time points and the 50% tissue culture infective dose (TCID_50_) was measured by Reed and Muench method [34].

### Determination of the viral L gene expression by qRT-PCR

To detect whether gga-miR-1603 and gga-miR-1794 affect the viral L gene expression at the mRNA level, DF-1 cells transfected with indicated miRNA oligonucleotides were inoculated with different NDV isolates for 24 h. Then, the RNAs were extracted using TRIzol Reagent (TransGen Biotech, China) following the protocol supplied. Viral L gene expression in RNA extractions was measured by qRT-PCR using TransScript Green One-Step qRT-PCR Super Mix (TransGen Biotech, China) on a LightCycler 480 machine (Roche, Switzerland). Viral L gene expression levels were normalized to those of *GAPDH* using the 2^−ΔΔCt^ method. All primers used are presented in Table 3.

**Table 3.** Primers used for qPCR detection of viral L gene

Primers	Primer sequence (5′–3′)
La Sota L F	CTTTCAATATCCAGCGGGAAG
La Sota L R	GAGGAGCACTTCAAGTGCTGC
LX L F	TTTCTTTCAAGGGAATGGGGTC
LX L R	CTCCAATTAAGACAGTACTTTTGC
Mukerswar L F	TTGTCGCGTTGCCTGTATGGTA
Mukerswar L R	GCTGTATATGAAGAACGTGTCTG
Herts/33 L F	CGACCGACTGTGATCTATCAAGAG
Herts/33 L R	ACCATCTCTGGGGAATCATCTG
NDV-P05 L F	GGATTCGCATACGGTGCCGT
NDV-P05 L R	GCGCACATCTGGCTCCTGAC
Kuwait L F	GGCATTGAGGGGTTATGCCAG
Kuwait L R	CGAGGAGTCATCTGACCTTACC
ZJ1 L F	TGGATTCATCTTAGGCTGATGGAC
ZJ1 L R	CATGCAGGCAACTCGACAAT
GAPDH F	GAGGGTAGTGAAGGCTGCTG
GAPDH R	CACAACACGGTTGCTGTATC

### Statistical analysis

All data are presented as the mean ± SD from three independent experiments. Statistical analyses were performed by one-way analysis of variance followed by Bonferroni’s multiple comparison tests using GraphPad Prism 5 (GraphPad Software, USA), and a *P* value < 0.05 indicated statistically significant.

## Results

### Prediction of candidate miRNAs targeting NDV antigenomic RNA

Evidence is accumulating that host miRNAs can regulate viral replication by directly binding to the viral genome, in addition to indirect interactions with host mRNAs. To investigate whether *Gallus gallus* miRNAs are involved in mediating host and NDV direct interactions, viral antigenomic RNA obtained from seven genotypes was used to predict potential miRNA targets with RegRNA 2.0. As shown in Table 4, approximately 150 miRNAs were predicted to target viral antigenomic RNA of each strain used in this study. As expected, the number of predicted miRNAs for each viral gene was positively correlated with gene length, and among these, the viral L gene harbored the most abundant miRNA-binding sites.

**Table 4.** Distribution of predicted miRNAs in each viral gene

		Number of predicted miRNA for each gene						
NDV strain	Accession Number	NP	P	M	F	HN	L	Total
LX	KF494201	23	27	12	29	32	69	170
La Sota	AF077761	18	29	9	27	23	61	142
Mukteswar	EF201805	17	29	13	29	29	68	166
Herts/33	AY741404	17	19	9	36	24	64	154
NDV-P05	HM117720	18	14	12	26	32	69	154
Kuwait	MK978147	21	21	12	19	30	53	154
ZJ1	AF431744	19	20	8	23	29	59	153
JS 5/05	JN631747	20	19	10	34	26	67	162

Subsequently, the predicted results of different strains were intersected to screen broad-spectrum miRNAs. As shown in Table 5, a total of 11 miRNAs was obtained after intersection processing. Interestingly, gga-miR-1671 was found to target more than one gene, including viral NP, HN, and L. In addition, no miRNA-binding site was found in the viral M gene.

**Table 5.** Details of broad-spectrum miRNAs targeted different genotypes of NDV strains

Viral Gene	miRNA	Binding Site (nt)^a^	Score	MFE (Kcal/mol)
NP	gga-miR-551	620 ~ 640	159	−30.0
	gga-miR-1671	1342 ~ 1363	168	−32.1
P	gga-miR-1574-5p	2443 ~ 2465	156	−28.4
	gga-miR-1658-3p	2139 ~ 2164	160	−32.9
	gga-miR-1597	2979 ~ 2999	154	−27.1
F	gga-miR-1610	4962 ~ 4982	158	−27.8
	gga-miR-1735	4712 ~ 4734	161	−29.1
HN	gga-miR-1671	7721 ~ 7743	167	−34.3
	gga-miR-1695	7152 ~ 7172	160	−26.9
L	gga-miR-1671	10,837 ~ 10,859	156	−30.2
	gga-miR-460b-5p	11,420 ~ 11,440	154	−26.8
	gga-miR-1603	8611 ~ 8634	169	−32.0
	gga-miR-1794	14,490 ~ 14,514	174	−31.4

^a^Position of miRNA-binding site was counted by JS 5/05.

### gga-miR-1603 and gga-miR-1794 directly target the viral L gene of La Sota

**Figure 1.**
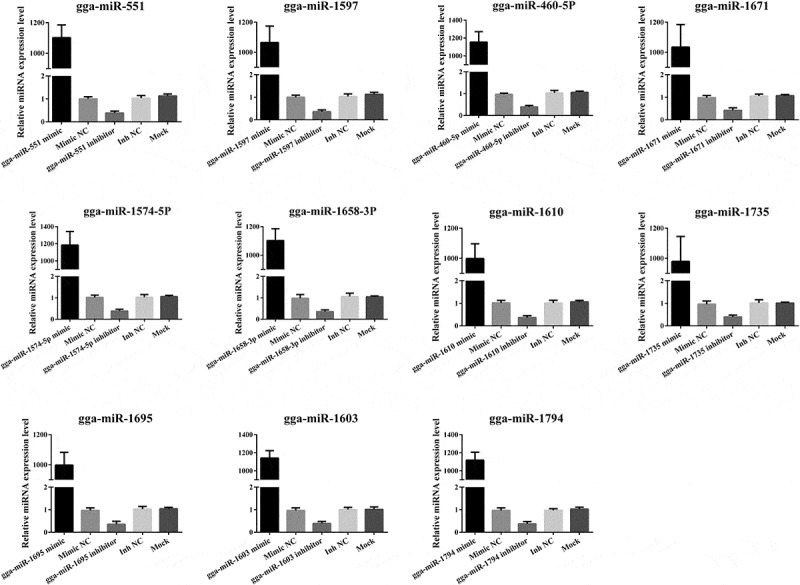
**Detection of miRNA oligonucleotide efficiency by qRT-PCR assay**. The indicated miRNA oligonucleotides (100 nM) were transfected into DF-1 cells for 24 h and untreated DF-1 cells were used as a blank control. The expression level of miRNAs was measured by qRT-PCR and normalized to 5S rRNA levels

**Figure 2.**
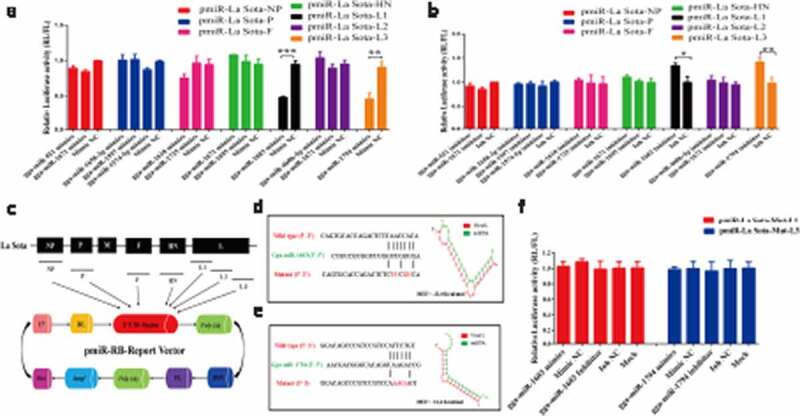
**Overall validation of potential miRNAs targeting antigenomic RNA of the La Sota strain by dual-luciferase arrays**. (a, b) Seven fragments of the La Sota antigenome were cloned into the luciferase reporter vector and then co-transfected with their corresponding predicted miRNA mimics (a) or inhibitors (b) before a dual-luciferase assay was performed. The construction strategy is shown in (c). The potential binding sites for gga-miR-1603 and gga-miR-1794 in the viral L gene are shown in (d) and (e), respectively, and the mutated pattern for the construction of pmiR-La Sota-Mut-L1 and pmiR-La Sota-Mut-L3 is indicated in red. (f) pmiR-La Sota-Mut-L1 or pmiR-La Sota-Mut-L3 were co-transfected with indicated miRNA oligonucleotides and a dual-luciferase assay was performed at 24 hours post-transfection. The *Renilla* luciferase (RL) activities were normalized to the value of firefly luciferase (FL). * *P* < 0.05, ** *P* < 0.01, and *** *P* < 0.001

To investigate whether predicated miRNAs target antigenomic RNA of NDV, we synthesized miRNA oligonucleotides and detected the transfection efficiency. As determined by qRT-PCR, miRNA levels in DF-1 cells transfected with their corresponding miRNA mimics were dramatically increased (> 1000-fold), but were decreased by 30% in cells transfected with the miRNA inhibitor (Figure 1). Next, we preliminarily validated the interaction between these potential miRNAs and their corresponding viral genes of La Sota by transfecting DF-1 cells with pmiR-La Sota-NP, P, F, HN, L1, L2, and L3 and their corresponding miRNA oligonucleotides. As detected by dual-luciferase reporter assays, the results showed that the relative luciferase activities were only decreased when cells were co-transfected with gga-miR-1603 mimics and pmiR-La Sota-L1 or gga-miR-1794 mimics and pmiR-La Sota-L3, but remained unchanged in cells co-transfected with other combinations (Figure 2a). Meanwhile, the opposite effect was observed after the cells were co-transfected with the gga-miR-1603 inhibitor and pmiR-La Sota-L1 or the gga-miR-1794 inhibitor and pmiR-La Sota-L3 (Figure 2b).

For further verification, we constructed pmiR-La Sota-Mut-L1 and pmiR-La Sota-Mut-L3 by introducing mutations into the base-pairing regions of the corresponding wild-type luciferase reporters. As expected, the decreasing or increasing trend in the relative luciferase activity was completely abrogated in DF-1 cells co-transfected with pmiR-La Sota-Mut-L1 and gga-miR-1603 mimics or inhibitor, as was the case with the pair comprising pmiR-La Sota-Mut-L3 and gga-miR-1794 (figure 2f). Overall, these results demonstrated that among the 11 predicted miRNAs, gga-miR-1603 and gga-miR-1794 directly target different regions in the L gene of the La Sota strain.

### Conservation analysis of the predicted miRNA-binding sites

We next evaluated whether the binding sites of gga-miR-1603 and gga-miR-1794 were conserved in the L genes of multiple NDV genotypes. The complete antigenome sequences of 192 NDV strains were downloaded from Genbank and submitted to phylogenetic analysis using MEGA X with the maximum likelihood method. As shown in Figure S1, the phylogenetic tree showed that these strains belonged to 13 different genotypes of class II, among which genotype Ⅶ, Ⅵ, and ⅩⅪ comprised the majority. Then, all of these strains were used for conservation analysis. As shown in Table 6, the results revealed that target sites for the seed regions of gga-miR-1603 and gga-miR-1794 were highly conserved with a conservation rate of more than 94%. Meanwhile, 93.75% of NDV strains harbored conserved binding sites for both miRNAs.

**Table 6.** Conservation Analysis of predicted miRNAs binding sites

Genotypes	Number	Conservation rate for gga-miR-1603	Conservation rate for gga-miR-1794	Conservation rate for both miRNAs
Ⅵ	38	94.74%	100.00%	94.74%
Ⅶ	76	96.05%	97.37%	93.42%
ⅩⅪ	20	95.00%	100.00%	95.00%
Others	41	95.12%	97.56%	92.68%
unclassified	17	94.12%	94.12%	94.12%
Total	192	95.31%	97.92%	93.75%

### gga-miR-1603 and gga-miR-1794 directly target the L genes of different NDV genotypes

**Figure 3.**
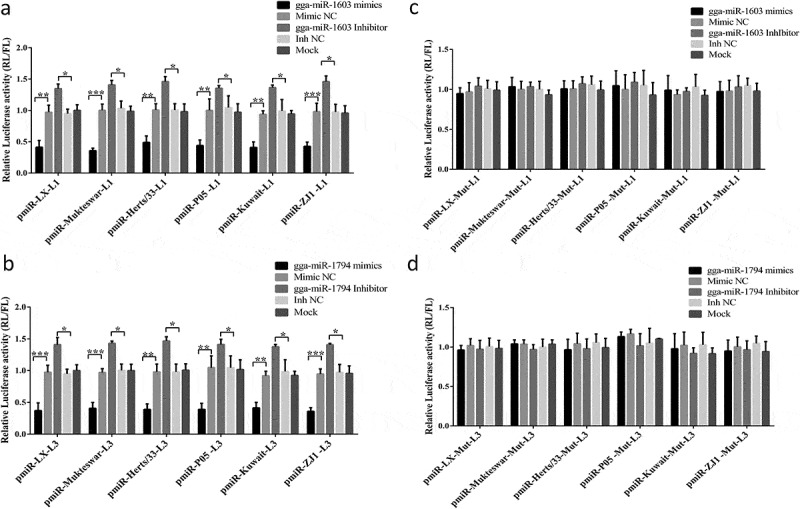
**Gga-miR-1603 and gga-miR-1794 directly target the viral L gene of multiple genotypes Newcastle disease viruses (NDVs)**. Wild-type dual-luciferase plasmids containing binding sites for gga-miR-1603 (a) or gga-miR-1794 (b) or mutated dual-luciferase plasmids with mutated sequences for the seed regions of gga-miR-1603 (c) or gga-miR-1794 (d) were co-transfected with corresponding miRNA oligonucleotides into DF-1 cells; then, cell lysates were harvested for dual-luciferase assays at 24 hours post-transfection. The *Renilla* luciferase (RL) activities were normalized to the value of firefly luciferase (FL). * *P* < 0.05, ** *P* < 0.01, *** *P* < 0.001

To experimentally explore whether the direct interaction between these two miRNAs and viral L genes is widespread among various genotypes of NDVs, the other six strains belonging to different genotypes were chosen as representatives to assess the binding ability of both miRNAs. As expected, the relative luciferase activity was markedly decreased in DF-1 cells co-transfected with gga-miR-1603 mimics and luciferase vectors containing the L1 region but increased in cells when the gga-miR-1603 inhibitor was transfected instead of mimics (Figure 3a). Additionally, similar results were observed in cells co-transfected with gga-miR-1794 mimics or the inhibitor and luciferase vectors containing the L3 region (Figure 3b). However, mimics or the inhibitor of these two miRNAs had no effect on the relative luciferase activity in the presence of a luciferase vector based on a mutant L gene lacking the gga-miR-1603 (Figure 3c) or gga-miR-1794 target sites (Figure 3d). Together, these results suggested that gga-miR-1603 and gga-miR-1794 directly target the L gene of multiple NDV genotypes.

### gga-miR-1603 and gga-miR-1794 negatively regulate viral L gene expression at both the protein and RNA levels

**Figure 4.**
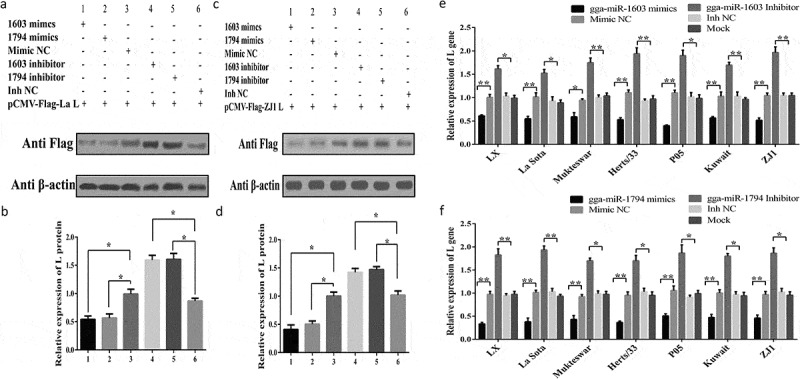
**Gga-miR-1603 and gga-miR-1794 negatively regulate viral L gene expression at both the protein and RNA levels**. (a, c) DF-1 cells were co-transfected with pCMV-Flag-La L (a) or pCMV-Flag-ZJ1 L (c) and indicated miRNA oligonucleotides. After 24 h, total protein in the cells was harvested for western blotting. The relative expression of L protein was analyzed using Image J software, and this is shown in (b) for the results of **(A)** and (d) for **(C)**. (e, f) DF-1 cells were transfected with indicated miRNA oligonucleotides of gga-miR-1603 (e) and gga-miR-1794 (f) for 24 h and then the cells were inoculated with different NDV isolates (0.1 MOI). Total RNA was extracted for qRT-PCR at 24 hpi. Viral L gene expression was normalized to GAPDH using the 2^−ΔΔCt^ method. * *P* < 0.05 and ** *P* < 0.01

To investigate whether and how gga-miR-1603 and gga-miR-1794 affect viral L gene expression, DF-1 cells transfected with gga-miR-1603 and gga-miR-1794 were infected with NDV strains or co-transfected with fusional expression plasmids; then, the mRNA and protein expression levels of the viral L gene were detected by qRT-PCR and western blotting, respectively. As shown in Figure 4a and 4b, expression of the viral L protein was substantially suppressed in gga-miR-1603- or gga-miR-1794-overexpressing cells, whereas the levels were increased markedly in cells transfected with the inhibitor of these two miRNAs. Similar suppression or promotion of L gene expression at the RNA level was observed when various NDV strains were used to infect mimic- or inhibitor-transfected DF-1 cells (Figure 4e & 4f). Together with our previous results, these results suggested that gga-miR-1603 and gga-miR-1794 inhibit expression of the L gene at both the protein and RNA levels by directly binding it.

### *gga-miR-1603 and gga-miR-1794 suppress NDV replication* in vitro

**Figure 5.**
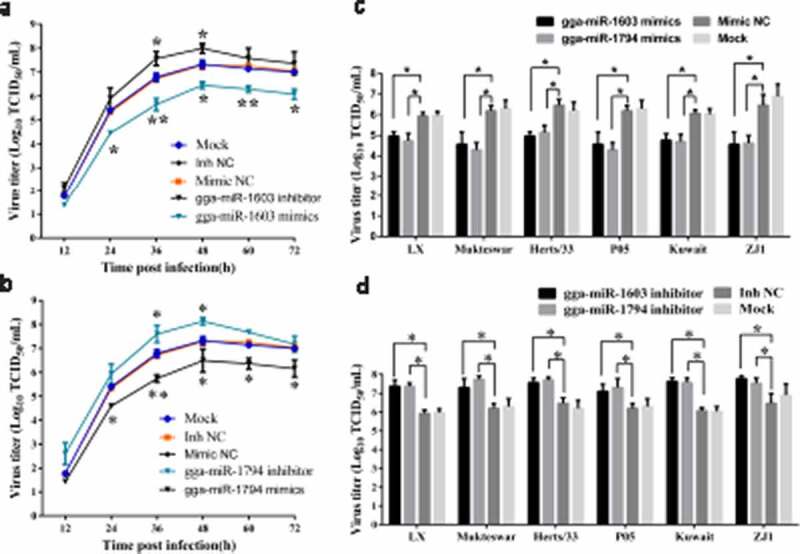
**Gga-miR-1603 and gga-miR-1794 suppress Newcastle disease virus (NDV) replication *in vitro***. (a, b) DF-1 cells were first transfected with miRNA oligonucleotides of gga-miR-1603 (a) or gga-miR-1794 (b) for 24 h, and then the cells were inoculated with the La Sota strain (0.1 MOI). The cellular supernatants were collected at indicated time points post infection and viral load was quantified based on TCID_50_. (c, d) DF-1 cells were transfected with miRNA mimics (c) or inhibitors (d) of gga-miR-1603 and gga-miR-1794 for 24 h. Then, the cells were infected with indicated NDV strains at an MOI of 0.1. Virus titers in cellular supernatants collected at 36 hpi were measured based on the TCID_50_. * *P* < 0.05 and ** *P* < 0.01

To detect the influence of these two miRNAs on viral replication, DF-1 cells transfected with miRNA oligonucleotides of gga-miR-1603 or gga-miR-1794 were infected with various NDV strains. Based on a determination of virus growth curves, the results indicated that mimics of gga-miR-1603 and gga-miR-1794 significantly decreased La Sota replication in DF-1 cells, whereas gga-miR-1603 and gga-miR-1794 inhibitors exerted the opposite effect (Figure 5a & 5b). Additionally, viral titers measured at 36 hpi for different NDV strains also revealed that these two miRNAs negatively regulated the replication of a variety of NDV strains *in vitro* (Figure 5c & 5d). These data indicated that gga-miR-1603 and gga-miR-1794 might have a broad-spectrum antiviral role during infection by multiple genotypes of NDVs.

### *NDV infection exerts no effect on gga-miR-1603 and gga-miR-1794 expression* in vitro

**Figure 6.**
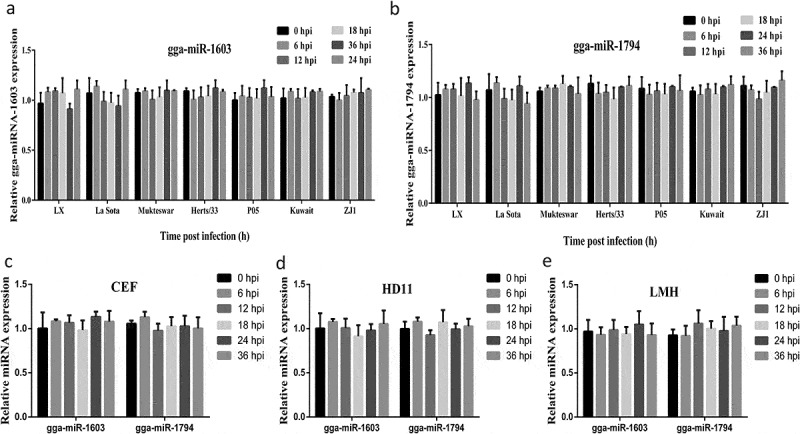
**Newcastle disease virus (NDV) infection exerts no effect on gga-miR-1603 and gga-miR-1794 expression *in vitro***. (a, b) DF-1 cells were inoculated with indicated NDV strains (0.1 MOI). The total miRNAs were extracted from cells collected at indicated time points post-NDV infection. Then, the expression levels of gga-miR-1603 (a) and gga-miR-1794 (b) were detected by qRT-PCR. **(C, E, F)** Three different avian cells lines, including CEF (c), HD11 (d), and LMH (e), were infected with the La Sota strain (0.1 MOI). The expression level of both miRNAs at different time points was measured using qRT-PCR. The relative expression of miRNAs was calculated by normalizing levels to 5S rRNA expression

Since gga-miR-1603 and gga-miR-1794 inhibited the replication of NDV, we wondered whether the virus would affect the expression of these two miRNAs. Therefore, the changes in abundances of both miRNAs in response to NDV infection were detected by qRT-PCR. The results indicated that expression of both miRNAs was not significantly changed throughout the detection period during the infection of DF-1 cells with various NDV strains (Figure 6a & 6b). Furthermore, we detected endogenous gga-miR-1603 and gga-miR-1794 expression levels in other avian cell lines, including CEF, HD11, and LMH, followed by infection with the La Sota strain. The results showed that infection by La Sota also had no influence on the expression of both miRNAs in all cell lines used in this study (Figure 6c–6e). In conclusion, these results suggested that NDV infection has no effect on gga-miR-1603 and gga-miR-1794 expression *in vitro*.

## Discussion

Increasing evidence has revealed that cellular miRNAs might play a critical role in the viral life cycle by directly binding the genomes or antigenomes of a broad range of viruses [35–37]. Here, to address whether miRNAs affect NDV replication directly, we screened and verified miRNA targets in antigenomic RNAs of various NDV strains. We demonstrated that gga-miR-1603 and gga-miR-1794 target two distinct regions of the viral L gene, located at 8611–8634 nt and 14,990–14,514 nt, respectively. Further conservation analysis and experimental validation indicated that these two miRNA binding sites are highly conserved and might function as effective antiviral targets to protect against NDV infection. Interestingly, these two conserved regions are both located in the coding region of the viral L gene, despite the fact that naturally occurring miRNA-binding sites are most commonly located in the UTRs of viral genomes [18]. This non-canonical binding pattern was also observed during the replication of some other viruses [38–40]. For example, gga-miR-21 negatively regulates IBDV replication *in vitro* by directly binding the coding region of viral VP1 gene [38]. We proposed that different from that in some other viruses, the UTRs of the NDV genome could be too short to allow for the presence of miRNA-binding sites, and thus, the coding region accounting for approximately 93% of the whole genome might function as targets of host miRNAs. However, further bioinformatic analysis of the commonality among the UTRs in the viruses indicated that a shared non-canonical binding pattern will be required to validate this speculation.

Two outcomes of interactions between an miRNA and its target have thus far been identified. Specifically, imperfect seed sequence complementarity generally leads to the inhibition of translation of targets with no influence on RNA abundance; however an exact seed sequence match usually results in the inhibition of target translation and eventually RNA degradation [41–43]. In this study, we found that gga-miR-1603 and gga-miR-1794 inhibited viral L gene expression at both the protein and RNA levels, even though only six nucleotides matched between the gga-miR-1794 seed region and viral L. Interestingly, we found that gga-miR-1603, for which the seed region is a perfect match to the viral L gene, exerts a more obvious effect on the RNA level of viral L gene than gga-miR-1794. These results might indicate that increasing the number of seed region matched sites in a viral gene could lead to increased RNA degradation. Further mutational analysis of miRNA-binding sites in the viral L gene will be helpful to clarify the regulatory mechanism of this imperfect binding pattern.

In this study, we also found that gga-miR-1603 and gga-miR-1794 negatively regulate NDV replication *in vitro*. The L protein, as the largest protein of NDV, assists in genomic RNA transcription and replication, thus regulating viral replication in host cells [44]. Given their inhibitory effects on the viral L gene, it is easy to imagine that the suppression of NDV replication is likely due to the direct interaction between these two miRNAs and the viral L gene. However, detecting replication dynamics based on a recombinant virus containing mutant binding sites will be needed to confirm this conclusion.

Meanwhile, the regulatory networks between miRNAs and host mRNAs are extremely complicated, as each miRNA could regulate the expression of many host mRNAs. Until now, no reports about the effects of these two miRNAs on host mRNAs have been available. Therefore, functional research on gga-miR-1603 and gga-miR-1794 with respect to cellular processes will help to further elucidate the mechanisms by which they regulate NDV replication.

A prior study revealed that NDV infection significantly promotes the expression of gga-miR-375 in chicken embryonic visceral tissues, and this miRNA directly targets the M gene of NDV, thus decreasing viral replication *in vitro* [45]. The secondary structure of this interaction predicted by RNA Hybrid software shows a high MFE (−23.4 Kcal/mol). However, our study failed to find this interaction, according to our prediction. This false negative is probably due to the strict screen criteria set for our prediction (MFE ≤ −25.00 Kcal/mol). This therefore indicates that deeper bioinformatic predictions will be beneficial to find more miRNAs that directly target the NDV genome.

MiRNAs can inhibit viral replication by directly binding to their genomes, whereas some viruses have also evolved to evade miRNA suppression through special mechanisms [36,46]. For example, miR-32 inhibits primate foamy retrovirus type 1 (PFV-1) replication by targeting the 3′ UTR of multiple viral genes. To overcome this inhibitory effect, Tas protein encoded by PFV-1 represses miRNA biogenesis, leading to reduced production of miR-32 *in vitro* [36]. Hsa-miR-23b suppresses Enterovirus 71 (EV71) replication by directly targeting the viral 3′ UTR conserved sequence; however, miR-23b is downregulated through an unknown mechanism due to EV71 infection, which finally prevents the direct interaction with the viral genome [46]. Furthermore, under strong selective pressure, RNA viruses might evolve to introduce mutations into the miRNA binding sites located in their genomes to evade miRNA mediated inhibition [35]. In our present study, we found that NDV infection had no effect on gga-miR-1603 and gga-miR-1794 expression *in vitro*. Interestingly, according to our previous deep sequencing data and qRT-PCR results in this study, we found that these two miRNAs exhibit a very low endogenous expression in different avian cell lines. Therefore, we surmise that host cells do not up-regulate these two miRNAs to inhibit viral replication during natural NDV infection. This might explain why the binding sites of these two miRNAs are maintained in the NDV genome during long-term evolution under selective pressure.

In summary, here, we provide evidence that gga-miR-1603 and gga-miR-1794 can directly bind to conserved regions of the NDV L gene and inhibit the replication of multiple NDV genotypes *in vitro*, suggesting that these two miRNAs have potential to be used as effective antiviral agents against NDV infection. Moreover, this study first discovered that the NDV L gene could regulate viral replication by providing miRNA binding sites, supplying new rationale for functional research on the L protein.

## Supplementary Material

Supplemental Material
