# Supplementary material for: gga-miR-1603 and gga-miR-1794 directly target viral L gene and function as a broad-spectrum antiviral factor against NDV replication
Source: Virulence. 2020 Dec 29;12(1):45–56. doi: 10.1080/21505594.2020.1864136 (PMC7781659; doi:10.1080/21505594.2020.1864136)
Supplement: Supplemental Material [file KVIR_A_1864136_SM8200.zip › supplement/Supplementary Materials.docx]

Table S1 Primers used for plasmids construction

| Primers | Primer sequences (5’-3’) |
| --- | --- |
| La Sota NP F | GTTTAAACACCAAACAGAGAATCCGTGAGTTAC |
| La Sota NP R | GCGGCCGCATTCCCTCGGCTCTGTTTTGATTG |
| La Sota P F | GTTTAAACTCCTCCCTCCCCCTGCT |
| La Sota P R | GCGGCCGCATCTTAAATGTAGCTAGATTAATTAC |
| La Sota F F | GTTTAAACCCCTTGCCAAATACAATCCTTTTA |
| La Sota F R | CTCGAGTGCGCCTTTTGCTTGTACATTAG |
| La Sota HN F | GTTTAAACAAGGCGCAACAAAAGACCTTATTA |
| La Sota HN R | GCGGCCGCATCAAGAGACTATTGACAAGGCTTA |
| La Sota L-1 F | GTTTAAACACGGGTAGGACATGGCGAGC |
| La Sota L-1 R | GCGGCCGCCGATCTAGCTGCAGCAAGTTGGATTGCAG |
| La Sota L-2 F | GTTTAAACTATCGAAGGATTATGCCAGAAGC |
| La Sota L-2 R | GCGGCCGCATCCGTATCTGCAAGTTGGTGTG |
| La Sota L-3 F | GTTTAAACCATTCATTCAAGGTTACATGCAGTG |
| La Sota L-3 R | GCGGCCGCCTTTTTTCTAACATAATATGATTAAA |
| LX L-1 F | GTTTAAACAGGATAGCACGGGTAGGACATG |
| LX L-1 R | GCGGCCGCCCAATTAAGACAGTACTTTTGCAGGT |
| LX L-3 F | GTTTAAACCCCACAATAGAGCTAATGAACATTC |
| LX L-3 R | GCGGCCGCACCAAACAAAGATTTGGTGAATGACA |
| Mukerswar L-1 F | GTTTAAACCCTGAATCTTGTCGATAGTCACTTG |
| Mukerswar L-1 R | GCGGCCGCCCATTAGTCTAAGATGAATCCACTCG |
| Mukerswar L-3 F | GTTTAAACAATTGAGTCACTCTTCCCAATGAC |
| Mukerswar L-3 R | GCGGCCGCACCAAACAAAGATTTGGTGAATGACATGA |
| Herts/33 L-1 F | GTTTAAACTCTTGTCGATAGTCACTTGGTTA |
| Herts/33 L-1 R | GCGGCCGCCAGCTTGACTGTCTGATATCTCC |
| Herts/33 L-3 F | GTTTAAACTCACTCTTCCCAATGACAACAACC |
| Herts/33 L-3 R | GCGGCCGCACCAAACAAAGATTTGGTGAATGACA |
| Kuwait L-1 F | GTTTAAACAACAACTTACAGAAGATGGCACG |
| Kuwait L-1 R | GCGGCCGCAGCTTGATTGTCTGATATCTCCAGTT |
| Kuwait L-3 F | GTTTAAACATCTCTAATTGAATCACTCTTCCCA |
| Kuwait L-3 R | GCGGCCGCACCAAACAAAGATTTGGTGAACGACA |
| ZJ1 L-1 F | GTTTAAACGATGTAAGGGAAAACAACCAACA |
| ZJ1 L-1 R | GCGGCCGCGCTGATTGATAGCATGAGCGAATAGT |
| ZJ1 L-3 F | GTTTAAACGCTCTTGGGTTTATCCCTAATTGA |
| ZJ1 L-3 R | GCGGCCGCACCAAACAGAGATTTGGTGAATGAC |
| La Sota L-1 Mut F | CTCTTCGTCAATTCCAGAATAACC |
| La Sota L-1 Mut R | TTCTGGAATTGACGAAGAGAGTTTGG |
| La Sota L-3 Mut F | CCGTCCATTCTGTGCGGAGA |
| La Sota L-3 Mut R | ACTCTAACGACGGACGGGCT |
| LX L-1 Mut F | CTCTTCGTCAATTCCAAGATAACCG |
| LX L-1 Mut R | TTGGAATTGACGAAGAGAGTCTGGTG |
| LX L-3 Mut F | CGTCGTTAGACTGCAGAGAGC |
| LX L-3 Mut R | CACTCTAACGACGGACGGGTTG |
| Mukerswar L-1 Mut F | CTCTTCGTCAATTCCAGGATAACCGG |
| Mukerswar L-1 Mut R | GGAATTGACGAAGAGAGTCTGGTGTA |
| Mukerswar L-3 Mut F | GTCGTTAGACTGCAGAGAGTTTAGT |
| Mukerswar L-3 Mut R | GCACTCTAACGACGGACAGGCT |
| Herts/33 L-1 Mut F | CTCTTCGTCAGTTCTAGGATAACCGG |
| Herts/33 L-1 Mut R | ACTGACGAAGAGAGTCTGGTGTACT |
| Herts/33 L-3 Mut F | GTCGTTAGACTGCAGAGAGTTTGGT |
| Herts/33 L-3 Mut R | CACTCTAACGACGGACAGGCT |
| Kuwait L-1 Mut F | CTCTTCGTCAATTCTAAGATAACCGGAG |
| Kuwait L-1 Mut R | AGAATTGACGAAGAGAGTCTGGTGC |
| Kuwait L-3 Mut F | GTCGTTAGACTGCAGAGAGTTTAGTG |
| Kuwait L-3 Mut R | CACTCTAACGACGGACAGGCTGC |
| ZJ1 L-1 Mut F | CTCTTCGTCAATTCCAAGATAACCGGA |
| ZJ1 L-1 Mut R | TTGACGAAGAGAGTCTGGTGCA |
| ZJ1 L-3 Mut F | GTCGTTAGACTGCGGAAAGTTTGG |
| ZJ1 L-3 Mut R | CACTCTAACGACGGACGGGCTG |
| La Sota L Flag F | GGGCCCATGGCGAGCTCCGGTCC |
| La Sota L Flag R | ACTAGTCTATTAATATGTGATTTTCGTTAAGAGTCA |
| ZJ1 L Flag F | GGGCCCATGGCGGGCTCCGGTCC |
| ZJ1 L Flag R | ACTAGTTTAGGAAGATAACTGATTGGCGTACGA |

Table S2 Sequences of miRNA oligonucleotides

| miRNA oligonucleotides | miRNA sequence (5′–3′) |
| --- | --- |
| gga-miR-551 mimics | sense: GCGACCCAUACUUGGUUUCAG |
|  | anti-sense: GAAACCAAGUAUGGGUCGCUU |
| gga-miR-1671 mimics | sense: GUGAGGACUGUUGAGUGGCCAAA |
|  | anti-sense: UGGCCACUCAACAGUCCUCACUU |
| gga-miR-1574-5p mimics | sense: GAGCUGUGGGUUGGUGUUGAUGG |
|  | anti-sense: UGACAAGGAGAAGUCACAGUU |
| gga-miR-1658-3p mimics | sense: GAGCUGUGGGUUGGUGUUGAUGG |
|  | anti-sense: AUCAACACCAACCCACAGCUCUU |
| gga-miR-1597 mimics | sense: UGAGGAGCUCUGCAAGCAUGCA |
|  | anti-sense: CAUGCUUGCAGAGCUCCUCAUU |
| gga-miR-1610 mimics | sense: UGGCUUGUGGUGGAACGGGCG |
|  | anti-sense: CCCGUUCCACCACAAGCCAUU |
| gga-miR-1735 mimics | sense: AGGGGCUUUGGGCAGCAUCUG |
|  | anti-sense: GAUGCUGCCCAAAGCCCCUUU |
| gga-miR-1695 mimics | sense: GAGCACAGUUUGGUCAUGGAGC |
|  | anti-sense: UCCAUGACCAAACUGUGCUCUU |
| gga-miR-460b-5p mimics | sense: UCCUCAUUGUACAUGCUGUGUG |
|  | anti-sense: CACAGCAUGUACAAUGAGGAUU |
| gga-miR-1603 mimics | sense: AGUGGUUGGUUUGGUGCUGUC |
|  | anti-sense: CAGCACCAAACCAACCACUUU |
| gga-miR-1794 mimics | sense: GCCAGAAUGGACAUGGGCAGCAA |
|  | anti-sense: GCUGCCCAUGUCCAUUCUGGCUU |
| miRNA mimics negative control | sense: UUCUCCGAACGUGUCACGUTT |
|  | anti-sense: ACGUGACACGUUCGGAGAAT |
| gga-miR-551 inhibitor | sense: UCACCAAGUAUGGGUCGC |
| gga-miR-1671 inhibitor | sense: ACUGGCCACUCAACAGUCCUCAC |
| gga-miR-1574-5p inhibitor | sense: ACUGACAAGGAGAAGUCACAG |
| gga-miR-1658-3p inhibitor | sense: UCAUCAACACCAACCCACAGCUC |
| gga-miR-1597 inhibitor | sense: UUCAUGCUUGCAGAGCUCCUCA |
| gga-miR-1610 inhibitor | sense: ACCCCGUUCCACCACAAGCCA |
| gga-miR-1735 inhibitor | sense: UCGAUGCUGCCCAAAGCCCCU |
| gga-miR-1695 inhibitor | sense: UAUCCAUGACCAAACUGUGCUC |
| gga-miR-460b-5p inhibitor | sense: ACCACAGCAUGUACAAUGAGGA |
| gga-miR-1603 inhibitor | sense: UCCAGCACCAAACCAACCACU |
| gga-miR-1794 inhibitor | sense: ACGCUGCCCAUGUCCAUUCUGGC |
| miRNA inhibitor negative control | Sense: CAGUACUUUUGUGUAGUACAA |

**Figure S1 Phylogenetic tree based on the complete F gene sequences (1662 nt) of NDVs.** The tree was constructed by the Maximum Likelihood method with 1000 bootstrap replicates in MEGA X. The class II complete F-gene dataset provided by Dimitrov et al were adopted as reference sequences. Sequences obtained in this study were shown in bold. Different colors of background indicated for different genotypes.
