# Supplementary figures and images for: gga-miR-1603 and gga-miR-1794 directly target viral L gene and function as a broad-spectrum antiviral factor against NDV replication
Source: Virulence. 2020 Dec 29;12(1):45–56. doi: 10.1080/21505594.2020.1864136 (PMC7781659; doi:10.1080/21505594.2020.1864136)

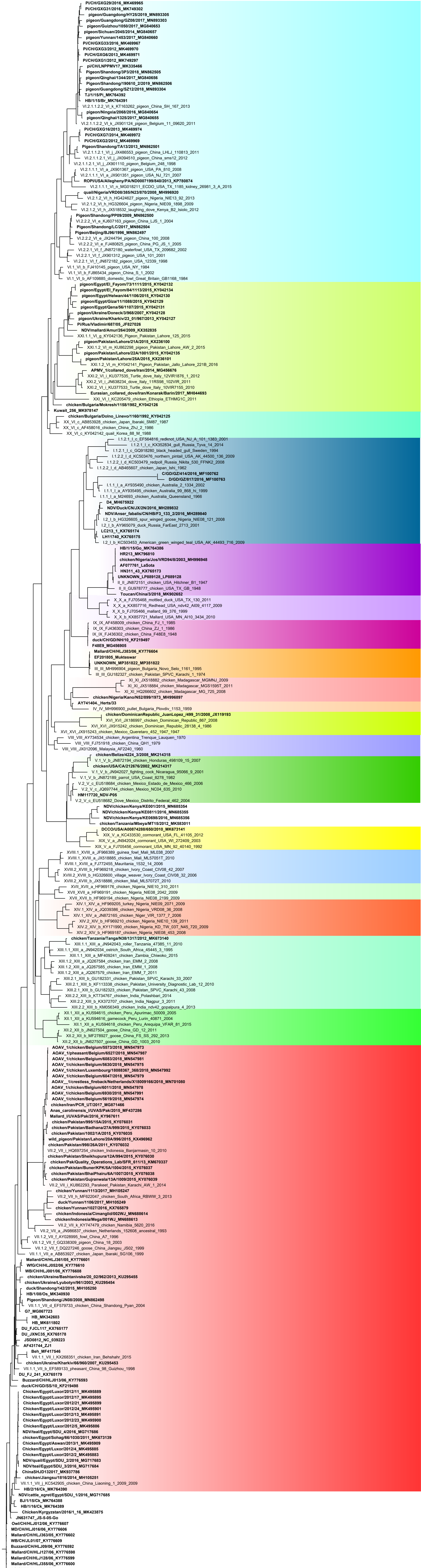

Supplement: Supplemental Material [file KVIR_A_1864136_SM8200.zip › supplement/Figure S1.pdf]
